# Gender-Dependent Alteration of Ca^2+^ and TNFα Signaling in *db*/*db* Mice, an Obesity-Linked Type 2 Diabetic Model

**DOI:** 10.3389/fphys.2019.00040

**Published:** 2019-02-07

**Authors:** Carmen Delgado, Ana-Maria Gomez, Magali Samia El Hayek, Gema Ruiz-Hurtado, Laetitia Pereira

**Affiliations:** ^1^ Instituto de Investigaciones Biomédicas “Alberto Sols” (CSIC-UAM)/CIBER-CV, Madrid, Spain; ^2^ INSERM UMR-S 1180, University of Paris-Sud, University of Paris-Saclay, Châtenay-Malabry, France; ^3^ Cardiorenal Translational Laboratory, Institute of Research i+12, Hospital Universitario 12 de Octubre/CIBER-CV, Madrid, Spain

**Keywords:** diabetic cardiomyopathy, TNFα, calcium, gender difference, *db*/*db* mice

## Abstract

Cardiovascular complications are the primary death cause in type 2 diabetes, where inflammation can play a role. We, and others, have previously shown that, in diabetic cardiomyopathy, cardiac dysfunction is associated with Ca^2+^ mishandling. It is possible that diabetic cardiomyopathy differently affects men and women, as the latter present higher risk to develop heart failure and a higher plasmatic level of the pro-inflammatory cytokine, tumor necrosis factor alpha (TNFα), than men. However, the gender-dependent regulation of Ca^2+^ signaling in diabetes and its relationship with TNFα signaling are still unclear. Here, we analyzed TNFα signaling pathway and its role in Ca^2+^ signaling dysfunction in male and female rodent models of type 2 diabetes linked to obesity (*db*/*db* mice) using confocal microscopy in freshly isolated cardiomyocytes. TNFα increased [Ca^2+^]_i_ transient amplitude and accelerated its decay without affecting SR Ca^2+^ load or Ca^2+^ spark frequency in cells from control mice. All TNFα effects on Ca^2+^ handling were prevented by the inhibition of the ceramidase and the phospholipase A2 (PLA2). While the plasmatic level of TNFα was similar in male and female *db*/*db* mice, only male *db*/*db* hearts over-expressed both TNFα converting enzyme (TACE) and the protective TNFα receptors 2 (TNF-R2). TNFα receptor 1 (TNF-R1) expression, involved in negative inotropic response of TNFα, was unchanged in both male and female *db*/*db* mice compared to controls. We found that male *db*/*db* mice cardiomyocytes presented a decrease in [Ca^2+^]_i_ transient amplitude associated to a drop of sarcoplasmic reticulum Ca^2+^ load, not seen in female *db*/*db* mice. Interestingly, sustained incubation with TNFα did not restored Ca^2+^ signaling alteration observed in male *db*/*db* mice but still induces an increase in Ca^2+^ spark frequency as seen in control littermates. In cardiomyocytes from female *db*/*db* mice, TNFα had no visible effects on Ca^2+^ handling. In conclusion, our study shows that the alteration of Ca^2+^ signaling and TNFα, seen in *db*/*db* mice, is gender specific presenting an increase in TNFα cardio-protective pathway in male mice.

## Introduction

Cardiovascular complications, such as coronary artery diseases, hypertension, and heart failure, are a leading cause of death in type 2 diabetes (Laakso, 1999; Bauters et al., 2003; Bell, 2007). Preclinical studies have shown that diabetic cardiac dysfunction, with depressed contraction and relaxation, results from dysregulation of metabolism, mitochondrial function, oxidative stress, and Ca^2+^ handling (Bugger and Abel, 2014). These knowledge result almost exclusively from male animal studies. However, in the clinical setting, the risk for developing cardiac diseases in diabetes is known to be gender specific (Galderisi et al., 1991; Rutter et al., 2003; Toedebusch et al., 2018). Indeed, the Framingham Heart Study showed that diabetic women present a 5.1-fold increased risk to develop heart failure than non-diabetic patients, whereas in diabetic men, this risk is only multiplied by 2.4 (Galderisi et al., 1991; Rutter et al., 2003). In addition, the hospital admission rate for cardiovascular diseases is higher in diabetic women compared to diabetic men. Yet, the gender differences in the alterations of cardiac cellular function in diabetes are unclear, notably regarding Ca^2+^ mishandling.

Ca^2+^ regulates contraction through the excitation-contraction coupling in cardiomyocytes. For each heartbeat, sarcolemmal L type Ca^2+^ channels open during the action potential, leading to Ca^2+^ influx that activates Ca^2+^ release from the ryanodine receptors (RyR) located at the sarcoplasmic reticulum (SR). This release of Ca^2+^ by the RyR (visualized as a [Ca^2+^]_i_ transient) activates contractile myofibrils to generate cardiomyocyte contraction. After the contraction, the Ca^2+^ is re-uptaken into the SR by the SERCA pump and extruded outside the cardiomyocytes mainly by the Na^+^/Ca^2+^ exchanger, resulting in cardiomyocyte relaxation. We and others have shown that, in animal models of type 2 diabetes linked to obesity, contractile dysfunction is associated with a decrease in the Ca^2+^ transient amplitude. This lower Ca^2+^ transient amplitude is associated to reduced L-type Ca^2+^ current density combined with downregulation of RyR expression (Belke et al., 2004; Pereira et al., 2006b, 2014). We found that these alterations may be different in male and female *db*/*db* mice (Pereira et al., 2014); however, the mechanisms remain unclear.

Clinical and preclinical studies pointed out an increase in plasmatic level of TNFα, in type 2 diabetes, notably in women (Yamakawa et al., 1995; Pereira et al., 2006a; Preciado-Puga et al., 2014). TNFα is an inflammatory cytokine commonly associated to infectious and non-infectious cardiomyopathy, such as viral myocarditis, congestive heart failure, and myocardial infarction. The level of TNFα seems correlated to the development of cardiac dysfunction (Feldman et al., 2000; Blum and Miller, 2001), and its over-expression leads to cardiac hypertrophy, fibrosis, arrhythmia, and dysfunction (Kubota et al., 1997; Kadokami et al., 2000; London et al., 2003). Yet, whether TNFα is a cause or a consequence of cardiac dysfunction is still under debate. The biological response of TNFα is mediated through two receptors, the TNFα receptor 1 (TNF-R1) and TNFα receptor 2 (TNF-R2). TNF-R1 activation is responsible for a cardiac negative inotropic response, whereas TNF-R2 mediates cardiac positive inotropic response (Meldrum, 1998). At the cellular level, TNFα regulates contraction either by direct regulation of Ca^2+^ signaling in acute condition or *via* iNOS activation in sustained conditions (Fernandez-Velasco et al., 2007). Still, whether TNFα activation positively or negatively alters the Ca^2+^ transient is quite controversial, and studies found either a decrease, an increase, or no effect on Ca^2+^ transient. Those discrepancies seem to depend on the animal model, the concentration of TNFα used, and the incubation time (Yokoyama et al., 1993; Goldhaber et al., 1996; Bick et al., 1997; Sugishita et al., 1999; Li et al., 2003; Zhang et al., 2005; Duncan et al., 2010; Greensmith and Nirmalan, 2013). In addition, whether the regulation of TNFα signaling in type 2 diabetic cardiomyopathy linked to obesity is gender specific remains unknown.

Considering all these controversial findings surrounding TNFα regulation of Ca^2+^ handling, we first studied the effect of TNFα on Ca^2+^ signaling in WT mice. Then, using the *db*/*db* mice, an animal model of type 2 diabetes with insulin resistance linked to obesity, we found that both Ca^2+^ and TNFα signaling underwent distinct alterations in male compared to female. Here, we found that male *db*/*db* mice presented a depressed Ca^2+^ transient associated with a lower SR Ca^2+^ load, not seen in female *db*/*db* mice. More interestingly, in male *db*/*db*, cardiomyocytes seem to put in place a protective mechanism to counteract those alterations by increasing the expression of cardio-protective TNF-R2 signaling pathway.

## Materials and Methods

### Cell Isolation

Experiments were carried out according to the ethical principles of the French Ministry of Agriculture and the European Parliament on the protection of animals. Ventricular adult cardiomyocytes were isolated from 8 weeks old male C56Bl6 mice, male and female 15 weeks old *db*/*db* (Janvier), and their control littermates (*db*/*+*). Mice were euthanized by intraperitoneal injection of sodium pentobarbital (100 mg/kg). Cardiac ventricular myocyte isolation was performed by standard enzymatic methods (collagenase type II, Worthington) using the Langendorff perfusion as previously described (Pereira et al., 2006b, 2007, 2012; Leroy et al., 2011; Ruiz-Hurtado et al., 2015). After isolation, cells were kept in 1 mM [Ca^2+^] for an hour prior experiments. Only rod-shaped cells and quiescent cells when unstimulated and excitable were used for the Ca^2+^ experiments.

### Measurements of Plasmatic TNFα

TNFα determination by ELISA Soluble TNFα concentration was determined in plasma samples from mice using commercial ELISA test (BIOTRAK, Amersham Life Science, Sweden).

### Confocal Microscopy

Ca^2+^ handling was recorded in freshly isolated ventricular adult cardiomyocytes loaded with the fluorescent Ca^2+^ dye, the Fluo-3 acetoxymethyl ester (Fluo-3 AM, Molecular Probes) at 5 μM diluted in a mixture of DMSO-pluronic acid 20%. A line scan across the longitudinal axis of the myocyte was performed to measure cardiomyocyte shortening. Cardiomyocyte shortening corresponds to the difference between cardiomyocyte length at rest and cardiomyocyte length during contraction (during electrical stimulation), as previously described (Fernandez-Velasco et al., 2009). Ca^2+^ transient, Ca^2+^ sparks, and SR Ca^2+^ load were recorded using confocal microscopy (Meta Zeiss LSM 510, objective w.i. 63×, n.a. 1.2) in line scan mode (1.54 ms) along the longitudinal axis of the cell. Ca^2+^ transients were evoked by field stimulation (1 Hz) applied through two parallel platinum electrodes. Spontaneous Ca^2+^ sparks were recorded in quiescent cells after Ca^2+^ transient recording. Ca^2+^ transient decay time corresponds to the kinetic of the relaxation phase due to the re-uptake of Ca^2+^ into the SR by the SERCA pump as well as the extrusion of Ca^2+^ by the Na^2+^/Ca^2+^ exchanger. Ca^2+^ transient decay time is calculated using a mono-exponential function to fit the Ca^2+^ transient decline phase. SR Ca^2+^ load was assessed by rapid caffeine application (10 mM) after 1 min pacing to reach the steady state. Parameters were studied with or without TNFα (1 h to 1 h 30 min) supplemented or not with a ceramidase inhibitor n-oleoylethanolamine (NOE, 5 μM) and a phospholipase A2 (PLA2) inhibitor (ATK, 10 μM) (Sigma-Aldrich). Fluo-3 AM was excited with an Argon laser (λ_ex_ = 488 nm), and emission was collected at wavelengths >505 nm. Image analysis was performed using homemade routines in interactive data language (IDL).

### Western-Blot Analysis

Adult ventricular homogenates were quickly frozen in liquid nitrogen and then placed in Tris solution (50 mmol/L, pH = 7.4) containing proteases and phosphatase inhibitors (10 μg/ml leupeptin, 10 μg/ml trypsin inhibitor, 2 μg/ml aprotinin, and 5 μM okadaic acid). Homogenization was performed on ice using a Politron. Homogenate was centrifuged at 18,925 g for 10 min at 4°C. Proteins were resuspended in Laemmli (5%) sample buffer, boiled (90°C for 5–10 min), and separated by sodium dodecyl sulfate polyacrylamide gel electrophoresis (SDS-PAGE) using 10% polyacrylamide gels. After separation, proteins were transferred to polyvinylidene fluoride membranes (Amersham Biosciences), and non-specific binding sites were blocked overnight at 4°C in 5% dried milk and Tris-buffer saline (TBS, pH = 7.4) and 0.01% Tween 20. Membranes were incubated overnight (at 4°C) for the rabbit polyclonal anti-TACE (1:300; Proscience) and the rabbit polyclonal anti-TNFR2 (H-202) (1:250; Santa Cruz), at room temperature for 1 h 30 min for the rabbit polyclonal anti-TNFR1 (H-271) (1:500; Santa Cruz). A secondary horseradish peroxidase-conjugated goat anti-rabbit IgG (Amersham Biosciences) was used in combination with an enhanced chemiluminescence detection system (SuperSignal West Pico Chemiluminescent Substrate, Pierce) to visualize the primary antibodies. Band densities were determined with a laser-scanning densitometer (HP-3970) and Quantity One software (BioRad SA). Protein loading was controlled by probing all Western blots with anti-GADPH antibody (1:4,000) (Ambion).

### Statistical Analysis

Results were expressed as mean ± SEM. Significance between two groups was determined using unpaired Student’s *t* test or non-parametric Mann-Whitney test. Data involving more than two groups were analyzed using either one-way ANOVA or two-way ANOVA as appropriate. We used GraphPad Prism 7 (GraphPad) for statistical comparison. Differences with values of *p* < 0.05 were considered significant.

## Results

### Sustained TNFα Exposure Increases Ca^2+^-Induced Ca^2+^ Release

**Figure 1:**
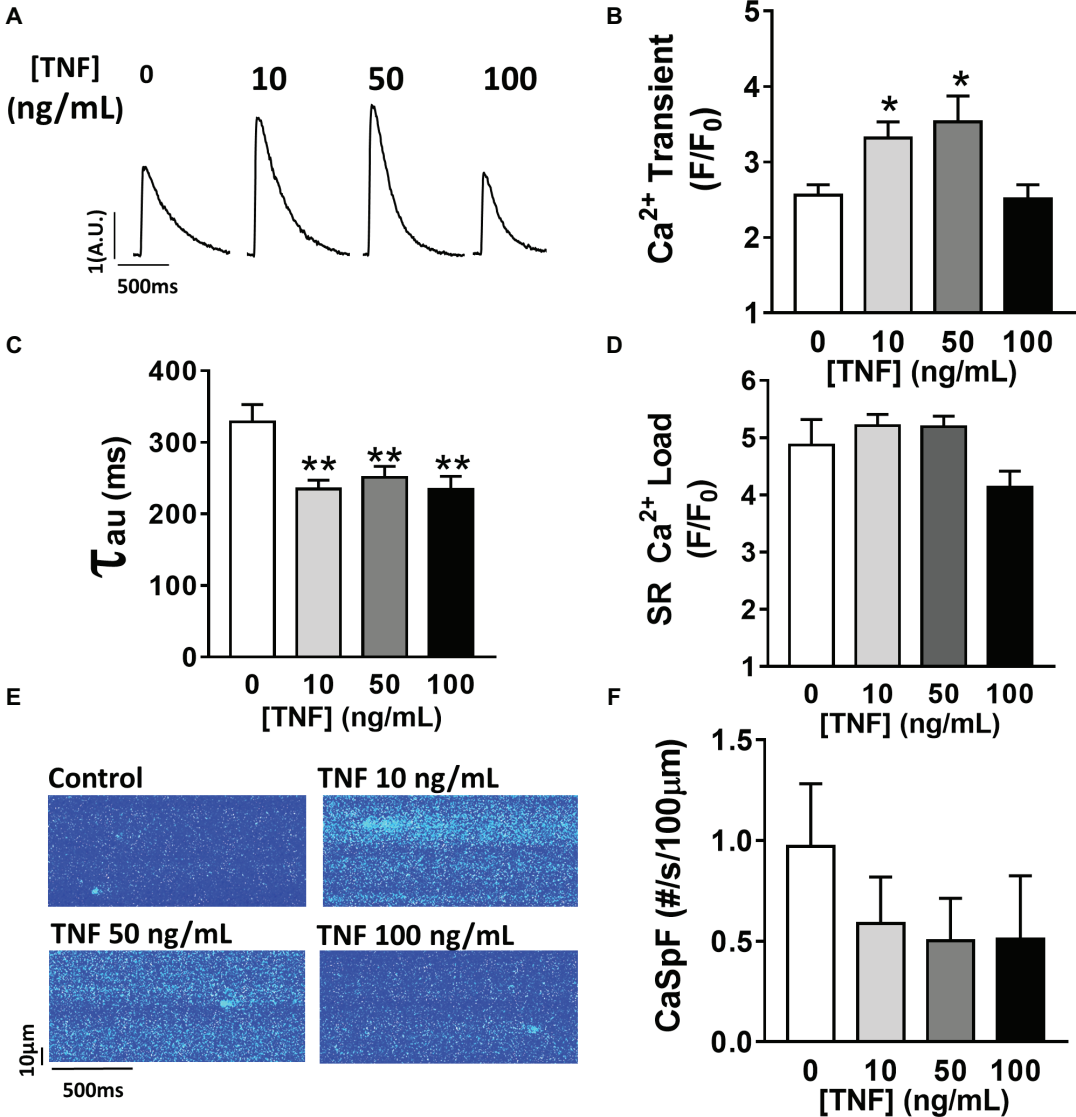
Positive inotropic effect of TNFα incubation. **(A)** Examples of Ca^2+^ transient recordings in freshly isolated cardiomyocytes (5 μM Fluo-3 AM) at baseline, at 10 ng/ml TNFα and 100 ng/ml TNFα. **(B)** Mean Ca^2+^ transient amplitude from cardiomyocytes at baseline (*n* = 22) and with incremental TNFα treatment (*n* = 21 for 10 ng/ml, *n* = 15 for 50 ng/ml, *n* = 10 for 100 ng*/*ml). **(C)** Ca^2+^ transient decay time (in ms) at baseline (*n* = 20) and with incremental TNFα treatments (*n* = 20 for 10 ng/ml, *n* = 15 for 50 ng/ml, *n* = 9 for 100 ng/ml). **(D**) Mean of sarcoplasmic reticulum (SR) Ca^2+^ load obtained by caffeine application after 1 min field stimulation in same conditions (respectively, *n* = 10, *n* = 11, *n* = 11, and *n* = 7). **(E)** Examples Ca^2+^ spark frequency (CaSpF) recording in freshly isolated cardiomyocytes at baseline, at 10 ng/ml TNFα and 100 ng/ml TNFα. **(F)** Mean of CaSpF (number of sparks (#) per second per 100 μm) obtained in same groups as in **(A)** (respectively, *n* = 19, *n* = 20, *n* = 15, and *n* = 7). *p < 0.05, **p < 0.01.

TNFα-mediated Ca^2+^ signaling regulation is quite controversial, which is probably due to protocol differences. Therefore, we first studied, in our experimental settings, the effect of sustained activation (1–1 h 30 min) of TNFα on Ca^2+^ handling parameters such as Ca^2+^ transient, Ca^2+^ spark frequency, and SR Ca^2+^ load (Figure 1). In our hands, 10 and 50 ng/ml TNFα treatment significantly increased Ca^2+^ transient amplitude (F/F_0_ of 3.1 ± 0.3 for 10 ng/ml, 3.5 ± 0.3 for 50 ng/ml vs. 2.5 ± 0.14 for baseline, *p* < 0.05). Moreover, TNFα significantly accelerated the Ca^2+^ re-uptake into the SR as shown by the faster SR Ca^2+^ transient decay time (Figures 1A,B) (~29% faster for 10 ng/ml and ~25% for 50 ng/ml, *p* < 0.01). This acceleration of Ca^2+^ re-uptake did not modified SR Ca^2+^ load (Figure 1D) and did not affect Ca^2+^ spark frequency (Figures 1E,F) at any concentration studied. However, 100 ng/ml of TNFα had no effects on either Ca^2+^ transient amplitude, Ca^2+^ spark frequency, or SR Ca^2+^ load. However, 100 ng/ml of TNFα still accelerated the Ca^2+^ transient decay (Figure 1C). These results clearly show that sustained TNFα activation mediates an increase in systolic Ca^2+^ release. Altogether, our results lean toward the idea of a positive inotropic effect.

### PLA2 and Ceramidase Mediate TNFα Regulation of Ca^2+^ Signaling

**Figure 2:**
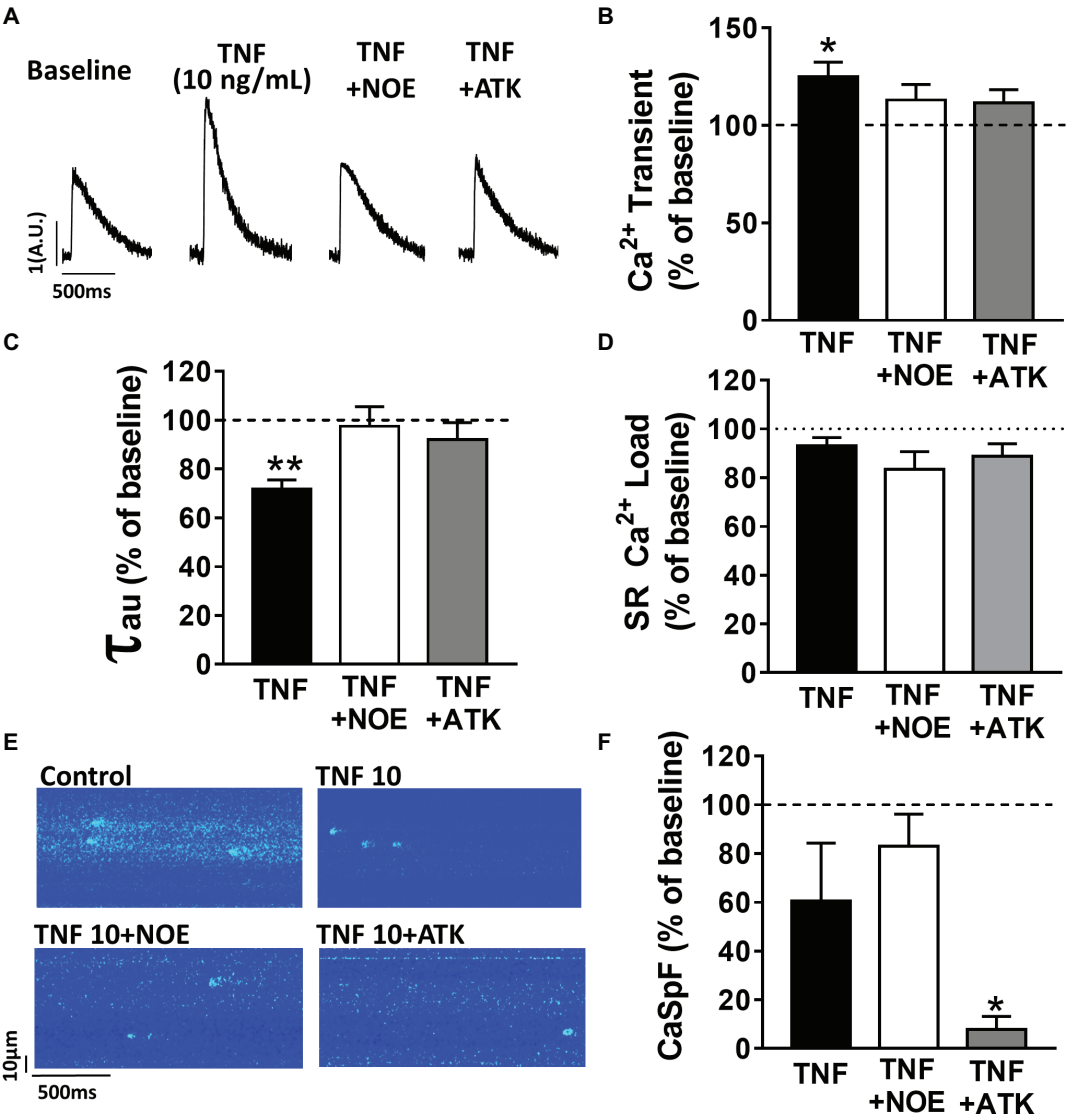
TNFα regulates Ca^2+^ signaling *via* the sphingosine pathway. **(A)** Representative Ca^2+^ transient examples obtained at baseline, at 10 ng/ml TNFα ± ceramide inhibitor (NOE) or phospholipase A2 inhibitor (ATK). **(B)** Percentage of effect on Ca^2+^ transient amplitude of TNFα treatment ±NOE or ATK (*n* = 20 for 10 ng/ml TNFα, *n* = 10 for TNFα+NOE, and *n* = 10 for TNFα+ATK). **(C)** Ca^2+^ transient decay time (in ms) in same groups (respectively, *n* = 19, *n* = 10, and *n* = 10). **(D**) Mean of SR Ca^2+^ load in the same groups (respectively, *n* = 11, *n* = 10, and *n* = 9). **(E)** Examples Ca^2+^ spark frequency recording in freshly isolated cardiomyocytes at baseline, at TNFα ± NOE or ATK. **(F)** Mean of CaSpF obtained in conditions (respectively, *n* = 20, *n* = 10, and *n* = 10). *p < 0.05, **p < 0.01.

Previous work has suggested that TNFα response is mediated by the sphingosine signaling pathway (Hofmann et al., 2003). To investigate the signaling pathway involved in TNFα regulation of Ca^2+^ signaling, we used a ceramidase inhibitor (5 μM NOE) and a PLA2 inhibitor (10 μM ATK). NOE fully prevented the increase of Ca^2+^ transient amplitude (Figures 2A,B) and the faster Ca^2+^ transient decay time induced by 10 ng/ml of TNFα (Figure 2C). NOE had no significant effects on neither the Ca^2+^ spark frequency nor the SR Ca^2+^ load (Figures 2D–F). Similarly, the phospholipase A2 inhibitor blunted all TNFα-mediated effects on the Ca^2+^ transient and the Ca^2+^ transient decay time (Figures 2B,C). As for NOE, ATK had no effect on SR Ca^2+^ load (Figure 2D). However, ATK, contrarily to NOE, did significantly reduce basal Ca^2+^ spark frequency. Altogether, those results suggest that TNFα alters Ca^2+^ signaling *via* the activation of the ceramidase and phospholipase A2 signaling pathway.

### Gender Differences in Upstream TNFα Signaling Pathway in Obesity-Linked Type 2 Diabetic Mice (*db*/*db*)

**Figure 3:**
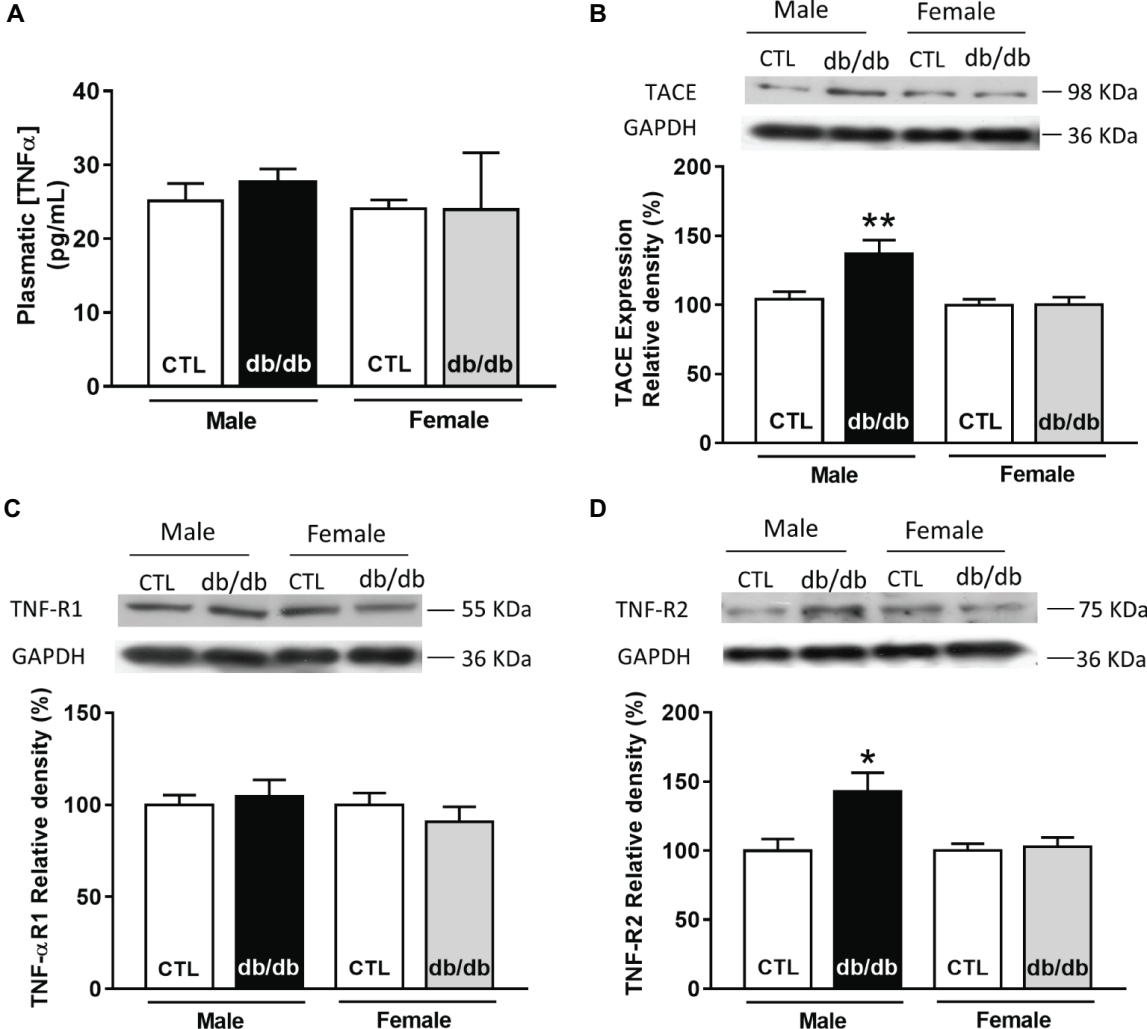
Gender-dependent alteration of the TNFα signaling pathway in type 2 diabetic mice (*db*/*db*). **(A)** Plasmatic level of TNFα obtained in male and female control and *db*/*db* heart tissue homogenates (male control: *n* = 5, male *db*/*db*: *n* = 7; female control and *db*/*db*: *n* = 8). **(B)** Representative Western-blot example of TACE (98 kDa) and the percent of relative density normalized by GAPDH signal from male and female control (*n* = 15, *n* = 17) and *db*/*db* (*n* = 14, *n* = 17) heart tissue homogenates. **(C)** Representative Western-blot example of TNF-R1 (55 kDa) expression and percentage of relative density normalized by GAPDH signal in male and female control (*n* = 16, *n* = 18) and *db*/*db* (*n* = 16, *n* = 18) heart tissue homogenates. **(D)** Representative Western-blot example of TNF-R2 (75 kDa) expression and percentage of relative density normalized by GAPDH signal in male and female control (*n* = 13, *n* = 13) and *db*/*db* (*n* = 13, *n* = 15) heart tissue homogenates. *p < 0.05.

Since plasmatic TNFα level is significantly elevated in type 2 diabetic patients, we first measured the plasmatic level of TNFα in male and female *db*/*db* mice. At 15 weeks old, *db*/*db* mice develop a type 2 diabetes linked to obesity with associated cardiomyopathy (Pereira et al., 2006b). Surprisingly, neither male nor female *db*/*db* mice presented an increase in their plasmatic level of TNFα compared to control (Figure 3A). Then, we measured the expression of key proteins involved in the TNFα signaling pathway, such as type 1 and type 2 TNFα receptors and the TNFα conversion enzyme TACE in both male and female *db*/*db* mice. Interestingly, TACE expression was significantly higher in male *db*/*db* mice compared to controls, whereas no change was detectable in the female group (Figure 3B). Moreover, while TNF-R1 receptor expression was unchanged in both *db*/*db* groups (Figure 3C), TNF-R2 in the *db*/*db* male group was significantly increased (Figure 3D). These results clearly suggest that in male *db*/*db* mice hearts, the TNF-R2, known to mediate a cardio-protective pathway, is over-expressed, probably to protect the heart from diabetic-induced stress.

### Gender Differences in Obesity-Linked Type 2 Diabetic (*db*/*db*) Ca^2+^ Mishandling

**Figure 4:**
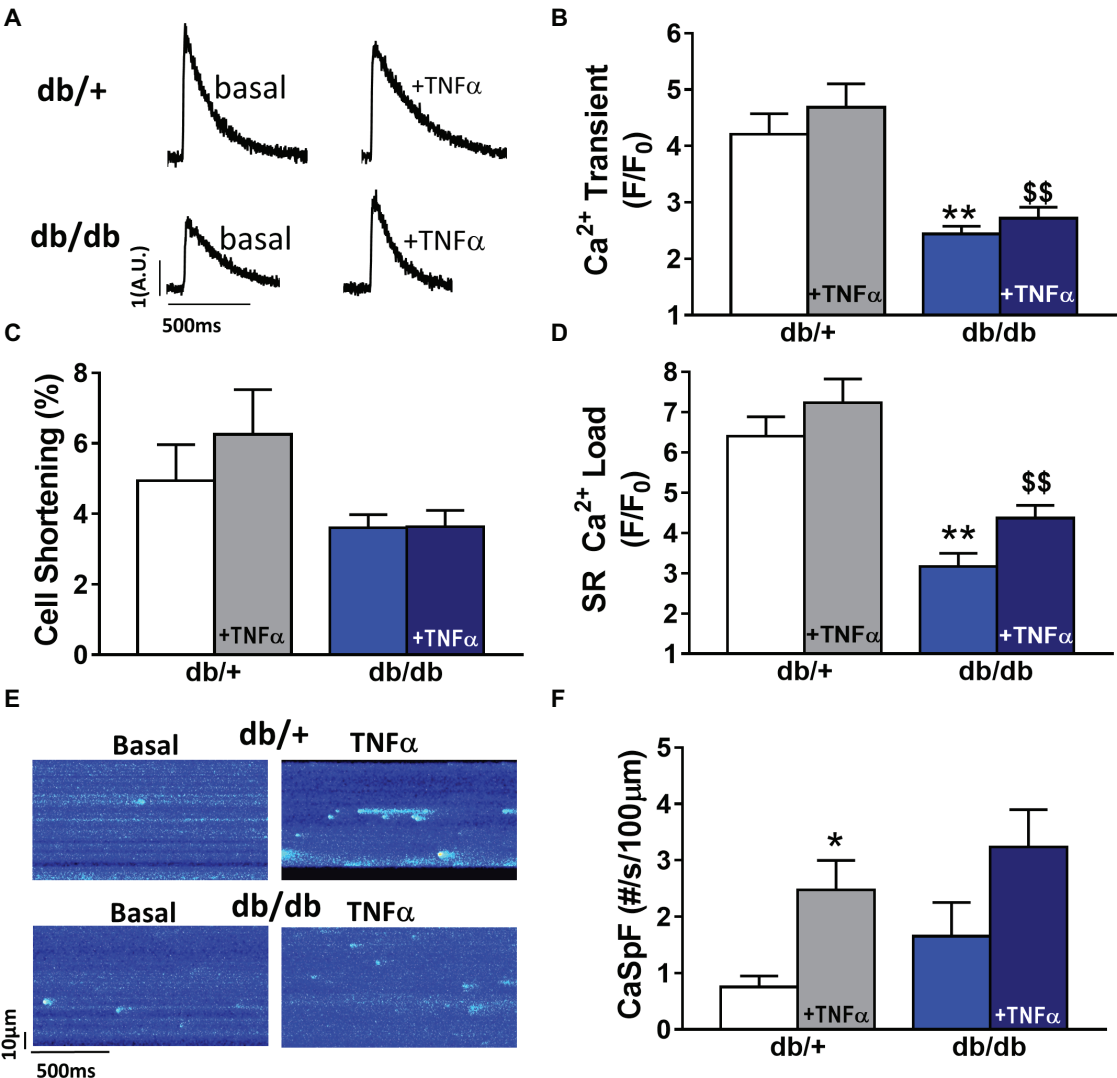
Ca^2+^ signaling is impaired in male type 2 diabetic mice. **(A)** Line scan images of Ca^2+^ transient recorded in male cardiomyocytes from *db*/+, *db*/*db* ± TNFα (10 ng/ml). **(B)** Mean of Ca^2+^ transient amplitude from *db*/*db* ± TNFα (*n* = 21 and *n* = 19) cardiomyocytes and their control littermates (*db*/+, *n* = 14 and *n* = 20). **(C)** Cell shortening measured in intact *db*/*db* ± TNFα (*n* = 13 and *n* = 15) and *db*/+ cardiomyocytes (*n* = 10 and *n* = 13) stimulated at 1 Hz. **(D)** Mean of SR Ca^2+^ load in intact *db*/*db* ± TNFα (*n* = 6 and *n* = 11) and *db*/+ cardiomyocytes (*n* = 10 and 11). **(E)** Examples Ca^2+^ spark frequency recording in freshly isolated cardiomyocytes at in *db*/+ and *db*/*db* with or without TNFα **(F)** Ca^2+^ spark frequency in the same groups (for *db*/*db*: *n* = 12 and 17, for *db*/+: *n* = 12 and *n* = 18) **p < 0.01 compared to *db*/+ and ^$$^ *p* < 0.05 compared to *db*/*db* without TNFα treatment.

**Figure 5:**
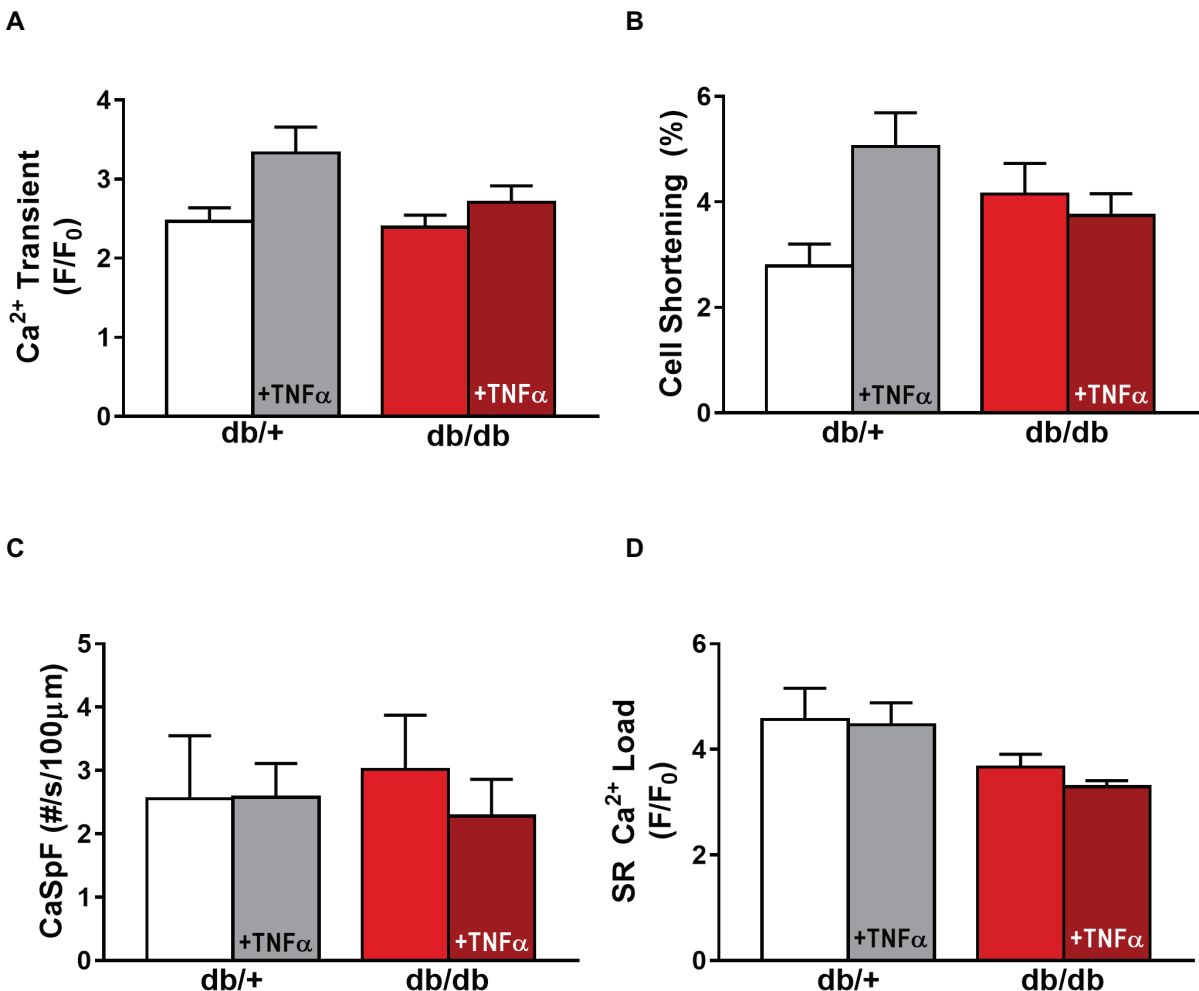
EC coupling is unchanged in type 2 diabetic female mice. **(A)** Mean Ca^2+^ transient amplitude from *db*/*db* ± TNFα (*n* = 27 and *n* = 23) cardiomyocytes and their control littermates (*db*/+, *n* = 11 and *n* = 18). **(B)** Cell shortening measured in intact *db*/*db* ± TNFα (*n* = 12 and *n* = 9) and *db*/+ cardiomyocytes (*n* = 8 and *n* = 14). **(C)** Ca^2+^ spark frequency in the same groups (for *db*/*db*: *n* = 9 and *n* = 12, for *db*/+: *n* = 10 and *n* = 17). **(D)** Mean of SR Ca^2+^ load in intact *db*/*db* ± TNFα (*n* = 17 and *n* = 18) and *db*/+ cardiomyocytes (*n* = 9 and *n* = 14). *p* = N.S.

In *db*/*db* mice, cardiac dysfunction has been associated with a decrease in SR Ca^2+^ transient amplitude and SR Ca^2+^ load (Belke et al., 2004; Pereira et al., 2006b, 2014). Here, we confirmed, in isolated cardiac myocytes from male *db*/*db* mice, that Ca^2+^ transient amplitude is significantly decreased (Figures 4A,B). This drop in Ca^2+^ transient amplitude (~51% lower than control, *p* < 0.01) is correlated with a drop in SR Ca^2+^ load (Figure 4D) (~51% lower than control, *p* < 0.01), which could explain the smaller (although not significant) cardiac cell shortening (Figure 4C). In our experimental conditions, Ca^2+^ spark frequency does not seem to be altered in *db*/*db* compared to control (*db*/*+*) (*p* = N.S.) (Figures 4E,F). In female *db*/*db* mice, the Ca^2+^ handling was similar in *db*/*db* compared to their control littermates (Figure 5). Indeed, all parameters such as Ca^2+^ transient amplitude (Figure 5A), Ca^2+^ spark frequency (Figure 5C), SR Ca^2+^ load (Figure 5D), and cell shortening (Figure 5B) were not significantly modified in freshly isolated cardiomyocytes in female *db*/*db* compared to control. In conclusion, we found a gender-specific alteration of Ca^2+^ handling in *db*/*db* mice, with lower SR Ca^2+^ release associated to a drop in SR Ca^2+^ load in male, not seen in female.

### Gender Differences of TNFα-Mediated Effect in Type 2 Diabetic (*db*/*db*)

Next, we compared TNFα regulation of Ca^2+^ signaling between male and female *db*/*db* mice. In male *db*/*db* mice, 10 ng/ml TNFα did not alter Ca^2+^ transient amplitude, cell shortening, nor SR Ca^2+^ load (Figures 4A–C,F). However, 10 ng/ml of TNFα similarly increased Ca^2+^ spark frequency in both control (~3.29 fold, *p* < 0.05) and *db*/*db* (1.5 fold, *p* = 0.06) (Figure 4D). In female control, the higher Ca^2+^ transient amplitude and cell shortening did not reach significance. Both female *db*/*db* and control had unchanged Ca^2+^ spark frequency. Those results suggest that, in 15 weeks old female *db*/*db*, the excitation-contraction coupling is unchanged compared to female control. Moreover, TNFα fails to show the effects found in male *db*/*db* (Figure 4D). Therefore, there are gender differences in Ca^2+^ mishandling and the underlying mechanisms in type 2 diabetes.

## Discussion

We have previously shown that cardiac dysfunction in type 2 diabetes is associated with cardiomyocyte Ca^2+^ mishandling, resulting from a decrease in the Ca^2+^ channels involved in the Ca^2+^-induced Ca^2+^ release process (RyR and L-Type Ca^2+^ channels) (Belke et al., 2004; Pereira et al., 2006b). Although TNFα is elevated in diabetic patient and animal model of diabetes (Yamakawa et al., 1995; Pereira et al., 2006a; Preciado-Puga et al., 2014), little was known about its role in cellular alteration, notably regarding the Ca^2+^ signaling pathway and gender specificity in animal model of diabetes linked to obesity. Here, we found a gender-specific alteration of Ca^2+^ and TNFα signaling in *db*/*db* mice, a common model of type 2 diabetes linked to obesity. Indeed, we found that male *db*/*db* mice, not female, presented the previously described Ca^2+^ mishandling with lower systolic Ca^2+^ release and SR Ca^2+^ load. More interestingly, we found that male and female *db*/*db* mice expressed differently TNF-R2, with an increased expression in male *db*/*db* mice that might reflect the activation of the TNFα cardio-protective TNF-R2-dependent pathway, not seen in female *db*/*db*.

### Cardiac Positive Inotropic Effect of TNFα

Discrepancies regarding the TNFα regulation of Ca^2+^ signaling are quite important in the literature with reported positive or negative ionotropic effect. For instance, in cat cardiomyocytes, short time exposure of TNFα reduced Ca^2+^ transient amplitude in response to a disruption of Ca^2+^ influx *via* L type Ca^2+^ channels leading to cellular shortening, supporting, then, a negative ionotropic effect of TNFα (Yokoyama et al., 1993). This negative inotropic effect of TNFα has been also described, in rabbit and guinea pigs, with TNFα-induced impaired cellular shortening cardiomyocytes mediated by NO dependent but Ca^2+^ independent (Goldhaber et al., 1996; Sugishita et al., 1999). However, various studies performed in rodents have shown that TNFα can lead to inotropic positive effects (Bick et al., 1997; Greensmith and Nirmalan, 2013). Here, we found that TNFα treatments (10 and 50 ng/ml) induced a time and concentration-dependent effect leading to a significant increase in Ca^2+^ transient amplitude between 1 h and 1 h 30 min suggesting a positive inotropic effect. Our results are in concordance with Bick et al. study (Bick et al., 1997), who have found that TNFα incubation increases Ca^2+^ transient and cellular contraction in neo-natal cardiomyocytes. In adult rat cardiomyocytes treated with 50 ng/ml of TNFα (Greensmith and Nirmalan, 2013), Ca^2+^ transient amplitude and cellular shortening were also increased (Greensmith and Nirmalan, 2013). The absence of effect observed under 100 ng/ml of TNFα might be explained by its bimodal effect, as previously described in cardiomyocytes, depending on exposure time or dose (Amadou et al., 2002; Shanmugam et al., 2016). Then, 100 ng/ml TNFα or higher doses, and with prolonged exposure, is expected to induce negative inotropic effects on Ca^2+^ handling.

### In Mice Cardiomyocytes, TNFα Regulates Ca^2+^ Signaling *via* the Sphingosine and PLA2 Pathways

Previous studies have shown that TNFα produces myocardial effects (negative or positive inotropic effect) through different mechanisms such as PLA2 or sphingosine signaling pathway (Murray and Freeman, 1996; Oral et al., 1997; Liu and McHowat, 1998). Here, we found that exposure of TNFα (1 h to 1 h 30 min) mediates Ca^2+^ transient increase *via* the activation of both ceramidase (sphingosine precursor) and PLA2 (for arachidonic acid production). Sphingosine is commonly associated to short-term (within minutes) negative inotropic effect of TNFα (Oral et al., 1997). However, other studies have shown that ceramide enhanced SR Ca^2+^ release and SR Ca^2+^ re-uptake in adult ventricular myocytes (Liu and Kennedy, 2003). Those results are in line with our prevention of TNFα-mediated elevation of systolic Ca^2+^ release and Ca^2+^ transient decay time in cardiomyocytes treated with the ceramidase inhibitor NOE (Figures 2B,C). Moreover, inhibition of the PLA2 prevented TNFα-mediated increase in Ca^2+^ transient amplitude and SR Ca^2+^ transient decay time, suggesting that TNFα induces Ca^2+^ mishandling *via* PLA2-mediated phosphorylation of RyR. Indeed, 10 ng/ml of TNFα has been shown to increase Ca^2+^ transient amplitude as a result of PLA-2 mediated RyR PKA phosphorylation at serine 2,808 in wild-type mice of RASSF1A knock out (Mohamed et al., 2014). This PKA-dependent mediated effect of PLA-2/arachidonic acid on the RyR phosphorylation state perfectly explains why we observed a dramatic drop of Ca^2+^ spark frequency under the inhibition of the PLA-2 (Figure 2F). In addition, TNFα also accelerates SR Ca^2+^ re-uptake reflecting an increase in SERCA pump activity as seen under PKA phosphorylation of phospholamban supporting the TNFα/PLA-2/PKA pathway. This mechanism is confirmed by the restoration of the TNFα-mediated acceleration Ca^2+^ transient decay time under ATK, the PLA-2 inhibitor (Figures 2B,C).

### Gender-Dependent Ca^2+^ Mishandling in *db*/*db* Mice, an Obesity-Linked Type 2 Diabetic Model

Type 2 diabetes is the most common form of diabetes. In western countries, 80% of type 2 diabetic patients have developed a diabetes linked to obesity resulting in severe glucose intolerance compared to lean type 2 diabetic patients (Schaffer and Mozaffari, 1996). Our study was performed in *db*/*db* mice, a model that recapitulates, in that sense, the human pathology. Indeed, the leptin receptor mutation of *db*/*db* mice impairs the satiety feeling and leads to obesity around 4–5 weeks of age, which is followed by diabetic state with hyperglycemia and insulin resistance (Coleman, 1978). In type 2 diabetes linked to obesity, cardiac dysfunction has been associated to Ca^2+^ mishandling and structural remodeling (Belke et al., 2004; Pereira et al., 2006b; Falcao-Pires and Leite-Moreira, 2012). Indeed, overall, animal models of type 2 diabetes present a reduced Na^+^/Ca^2+^ exchanger activity, and depressed Ca^2+^ transient linked to downregulation of Ca^2+^ channels, RyRs, and reduced SERCA activity (Netticadan et al., 2001; Zhong et al., 2001; Abe et al., 2002; Belke et al., 2004; Pereira et al., 2006b; Boudina and Abel, 2010). Here, our results show that those effects are recapitulated in male *db*/*db* mice (Figure 4), but not in female *db*/*db* mice. However, the gender-specific regulation in Ca^2+^ handling and/or β-adrenergic response has been previously described (Parks et al., 2014). Supporting this idea, we found that basal Ca^2+^ transient amplitude is lower in female control compared to male control cardiomyocytes. Although Parks et al. (2014) have shown that Ca^2+^ current, diastolic Ca^2+^, and SR Ca^2+^ load were similar between control male and female, basal cAMP level was lower in control female compared to control male due to higher PDE4B expression in female. These results are in line with our previous work showing that *db*/*db* female mice have reduced phosphorylation of the RyR, which reduce Ca^2+^ spark frequency and could explain the preserve SR Ca^2+^ load and Ca^2+^ transient seen in female *db*/*db* compared to *db*/*db* male. Our results are paradoxical compared to the higher risk to develop heart failure for type 2 diabetic women compared to diabetic men. This discrepancy could be explain as follow: the decrease in [Ca^2+^]_i_ transient in male *db*/*db* mice could be protective at long term, maybe by preventing Ca^2+^ toxic effects such as apoptosis or preserve ATP content by limiting the ATP expense in pumping Ca^2+^ (Javorkova et al., 2010; Parks et al., 2014). Future studies will be needed to confirm this hypothesis.

### Gender Dependent Alteration of Molecular TNFα Signaling Pathway in *db*/*db*

To our knowledge, plasmatic TNFα level parallels the degree of cardiac dysfunction in diabetic patients. In the *db*/*db* mice, we did not observe any changes in the plasmatic level of TNFα compared to control. Even though circulating TNFα is unchanged, male *db*/*db* mice present an increase in TACE expression suggesting a paracrine elevation of TNFα in the heart. Surprisingly, despite cardiomyocyte treatment with 10 ng/ml of TNFα, a concentration within the *in vivo* range measured under stress and injury (Bitterman et al., 1991), TNFα did not induce an increase in Ca^2+^ transient amplitude or decay time in *db*/*db*, as seen in C57Bl6 mice (Figures 4B,C). One explanation could be that in *db*/*db* control littermate strain background (C57BKS/J strain), TNFα is not as effective as in C57Bl6 strain. Indeed, genetic background, such as between C57BL6/J and C57BL6/N, has been shown to influence cardiac phenotype and propensity to develop cardiomyopathies (Tian et al., 2011; Simon et al., 2013). This could also explain the ineffective response of TNFα in female control and *db*/*db* mice (Figure 5). Although TNFα activation has been linked with oxidative stress, no gender-specific difference in cardiomyocytes redox state at baseline or during pathology has been observed (Ren, 2007; Bell et al., 2015). Another possibility could be that in male *db*/*db*, the dramatically reduced SR Ca^2+^ load would prevent the high Ca^2+^ systolic release induced by TNFα probably due to the phosphorylation of the RyR *via* the activation of PLA2. Indeed, we found in the presence of TNFα an increase in Ca^2+^ spark frequency in both *db*/*+* and *db*/*db* mice reflecting an elevated diastolic RyR opening resulting from RyR phosphorylation by PKA previously described in male *db*/*db* (Pereira et al., 2014). Interestingly, in male *db*/*db* mice, the TNF-R2 was overexpressed, which is known to exert cardio-protective effects *via* the activation of NF-κB (Burchfield et al., 2010). Indeed, in liver, TNFα inhibits PDE3 expression elevating cAMP level and PKA activation (Ke et al., 2015). This activation of PKA could explain, in cardiomyocytes, the elevation of Ca^2+^ spark frequency in male *db*/+ cardiomyocytes treated with TNFα (Figure 4F). Moreover, TNF-R2 is known to be involved in positive cardiac inotropic effect (Defer et al., 2007). As a result, [Ca^2+^] overload was prevented and Ca^2+^ transient increased leading to an increase in inotropic response. The over-expressed TNF-R2 in a male *db*/*db* appears as an attempt to counteract the already present Ca^2+^ mishandling to protect from cardiac dysfunction. Indeed, prolonged activation of the TNF-R2 pathway in the *db*/*db* male cardiomyocytes could then activate phosphorylation of excitation-contraction coupling key proteins, such as phospholamban, to restore Ca^2+^ transient and cardiomyocytes contraction.

In conclusion, we found for the first time that both Ca^2+^ and TNFα signaling are altered only in male type 2 diabetic mice, whereas female does not seem to be affected. Although this study has several limitations in the interpretation such as non-comparable hormonal state between female *db*/*db* mice and diabetic women, lower effect of TNFα in *db*/*+* than C57BL6 control, we still clearly show that male *db*/*db* mice develop Ca^2+^ mishandling leading to impaired contraction already at a young age, while woman seemed to be protected. Moreover, we found that male *db*/*db* mice put into place a protective mechanism to counteract those negative effects by over-expressing TNF-R2 cardio-protective signaling pathway.

## Data Availability

The datasets generated for this study are available on request to the corresponding author.

## Ethics Statement

The study was carried out in accordance to the ethical principles of the French Ministry of Agriculture and the European Parliament on the protection of animals. The protocol was approved by the French Ministry of Agriculture and Bioethical Committee of the CSIC following recommendation of the Spanish Animal Care and the European Parliament on the protection of animals.

## Author Contributions

CD and AG conceived and designed the project, supervised the data acquisition and participated in analysis. LP and GR performed most of the experiments and analyses. LP interpreted the data and wrote the first draft of the manuscript. MS participated in the figure preparation. All authors have edited the manuscript.

### Conflict of Interest Statement

The authors declare that the research was conducted in the absence of any commercial or financial relationships that could be construed as a potential conflict of interest.

## Abbreviations

ATK: arachidonyl trifluoromethyl ketone
TNFα: tumor necrosis factor alpha
TNF-R1: TNFα receptor 1
TNF-R2: TNFα receptor 2
KO: knock-out
NO: nitric oxide
NOE: n-oleoylethanolamine
o.i.: oil immersion
PKA: protein kinase A
PLA2: phospholipase A2
RyR: cardiac ryanodine receptor
SR: sarcoplasmic reticulum
SERCA: sarco/endoplasmic reticulum Ca^2+^-ATPase
TACE: TNFα converting enzyme.
